# Ketamine induces apoptosis in lung adenocarcinoma cells by regulating the expression of CD69

**DOI:** 10.1002/cam4.1288

**Published:** 2018-02-17

**Authors:** Xuhui Zhou, Peihong Zhang, Wei Luo, Lei Zhang, Rong Hu, Yu Sun, Hong Jiang

**Affiliations:** ^1^ Department of Anesthesiology Shanghai Ninth People's Hospital Shanghai Jiao Tong University School of Medicine Center for Specialty Strategy Research of Shanghai Jiao Tong University China Hospital Development Institute Shanghai 200011 China

**Keywords:** Apoptosis, CD69, ketamine, lung adenocarcinoma

## Abstract

Ketamine, an anesthetic, analgesic, or sedative, is widely used for the treatment of cancer pain. Recently, ketamine has been also reported to be tumor repressor for inhibiting proliferation, invasion, and migration, and inducing apoptosis in many cancers. However, whether ketamine can induce the apoptosis of lung adenocarcinoma (LUAD) and which downstream molecular mediates its function remain largely unknown. A LUAD cell line A549 was incubated with ketamine at 0, 1, 10, and 100 *μ*mol/L for 24 h. Trypan blue staining was used to detect the cell viability. Flow cytometry (FACS) was applied to evaluate cell apoptosis proportion. The expression of CD69 was quantitated by western blotting. Ketamine induced the A549 cell apoptosis in a concentration‐dependent manner. CD69 was downregulated in LUAD patients’ cancer tissue compared with the normal tissue. CD69 can be upregulated in ketamine treating A549 cells and induce the A549 cell apoptosis. Rescue experiment showed that downregulation of CD69 significantly blocked the function of ketamine on inducing apoptosis. Taken together, our results demonstrated that ketamine induced LUAD cells apoptosis by upregulating the CD69 expression. This study suggests that the ketamine can be potential drug for LUAD treatment, and the ketamine/CD69 signaling may be the new potential therapeutic target LUAD therapy.

## Introduction

Lung cancer is the leading cause of cancer‐related deaths worldwide. Non–small cell lung cancer (NSCLC) accounts for 85% of all lung cancers and has a 5‐year survival rate of 15%. Lung adenocarcinoma (LUAD) is one of the most common subtypes of NSCLC and accounts for approximately 80% of NSCLC cases 1, 2. Approximately 70% of patients with lung cancer present with locally advanced or metastatic disease at the time of diagnosis, and there is considerable toxicity associated with current chemotherapies 3. Rapid growth with little apoptosis, invasion, and migration are the most malignant characteristics of LUAD cells. The unrestricted growth leads to tumor enlargement, compression of the peripheral organs, and even invasion and metastasis. An effective strategy to treat malignant tumors is by inhibiting tumor cell growth 4. Thus, it is important to investigate the regulatory mechanisms of LUAD cell apoptosis. Ketamine, an NMDA (*N*‐methyl‐d‐aspartate) receptor antagonist, is widely used as an anesthetic, analgesic, or sedative in various clinical settings: for example, it is used as pain relief during the terminal stage of cancer 5, 6. In a series of animal experiments, ketamine has been shown to promote neuronal apoptosis and induce cell death in neurons and neural stem progenitor cells 7, 8, 9. Moreover, our previous study also revealed that repeated administration of ketamine can induce hippocampal neurodegeneration 10. Ketamine also regulates the proliferation and apoptosis of pancreatic cancer cells and HepG2 cells 11, 12. However, the effects of ketamine on regulating LUAD cell apoptosis and the underlying mechanism are not known. Apoptosis is a prominent mechanism associated with the induction of tumor remission 13. Suppression of apoptotic programs contributes to tumor initiation and reduces treatment sensitivity 14. Recent studies have shown that CD95 (also known as Fas and apo‐1), a member of the death receptor (DR) family, can initiate the extrinsic pathway of apoptosis 15. CD40, a costimulatory molecule, is expressed in diverse cell types and plays a critical role in regulating cell growth, cell proliferation, cell activation, and host antitumor responses 16. Moreover, CD69, a leukocyte and natural killer (NK) cell activation marker, belongs to the C‐type lectin‐like superfamily NK cell membrane receptors. This molecule forms homodimers in which the molecules are connected by disulfide bonds, and the dimer is a type II transmembrane protein. CD69 plays an important role in the activation of different leukocyte subsets and in the pathogenesis of tissue damage in different inflammatory conditions. CD69 has also been associated with apoptosis 17, 18. Higher expression of CD69 in T cells leads to the rapid apoptosis in vitro 19. Bcl‐2 mediates CD69‐induced apoptosis in human eosinophils 18. However, whether CD69 can regulate LUAD cell apoptosis and act as a therapeutic target remain unclear.

In this study, we found that ketamine induced A549 cell apoptosis in a concentration‐dependent manner. CD69 was downregulated in LUAD patient tissues compared with normal tissues. CD69 was upregulated in ketamine‐treated A549 cells and induced A549 cell apoptosis. Rescue experiments showed that the downregulation of CD69 significantly blocked the ketamine‐mediated induction of apoptosis.

Thus, our study elucidates the ketamine/CD69 signaling axis, which could be a potential target for future therapies.

## Materials and Methods

### Cell culture

A549 cells obtained from the American Type Culture Collection were cultured in RPMI‐1640 cell culture medium (Hyclone, USA) with 10% fetal bovine serum (FBS) (Gibco, USA) at 37°C, 5% CO_2_ atmosphere. The medium was also supplemented with 100 U/mL penicillin (Invitrogen, USA) and 100 *μ*g/mL streptomycin sulfate (Invitrogen, USA).

### Vector construction

#### Knockdown of CD69

The pLKO.1 plasmid containing shCD69 sequence was used to downregulate the expression of CD69. The shCD69 sequence was referenced by the previous study 20 and was as follows: 5′‐CCGGGCATGGAATGTGAGAAGAATTCTCGAGAATTCTTCTCACATTCCATGCTTTTTG‐3′.

#### Overexpression of CD69

The Fugw plasmid containing CD69 CDS sequence was used to overexpress the CD69. CDS fragment was obtained by PCR from the cDNA synthesized by reverse transcription PCR kit (Takara, Japan) from the total RNA of A549 cells. The primer sequences for PCR are as follows:

PF: 5′‐GGCGGATCCATGAGCTCTGAAAATTGTTTCGTAG‐3′ (BamH1 site).

PR: 5′‐GGCGAATTCTTATTTGTAAGGTTTGTTACATATCCAGTA‐3′ (EcoR1 site).

### Cell apoptosis analysis

Flow cytometry (FACS) was used to detect the apoptosis rates. The cells were treated by using apoptosis and necrosis assay kit (Beyotime, China).

### Trypan blue staining

Trypan blue solution (0.1%) was added into the cells suspension for 3–5 min. The number of dead cells (stained with blue) and living cells (without blue color) in total cells (counting 1000 cells per group) were then counted. The cells viability rate = number of living cells/(number of dead cells + living cells) × 100%.

### Western blot

Cells were lysed by 1X SDS sample buffer for electrophoresis. The protein was transferred onto the PVDF membrane (Bio‐Rad, USA) and incubated with primary antibody: CD69 antibody (sc‐373799, Santa Cruz, USA). GAPDH antibody (AP0063, Bioworld, China). The result was visualized by enhanced chemiluminescence (ECL) western blotting substrate (Thermo Fisher Scientific, USA).

### CD69 expression analysis in TCGA dataset

Whole mRNA expression data of patients’ samples were obtained from TCGA database (http://cancergenome.nih.gov/). We used RSEM value of genes for analysis. *T* test was used to test significance difference of CD69 expression between normal samples and cancer samples at different stages.

### Statistical analysis

For determining the statistical significance, we used the Student's *t* test or one‐way ANOVA for statistics. Values were presented as the mean ± SD (*P < 0.05, **P < 0.01, ***P < 0.001). GraphPad Prism was used to analyze experiment data (GraphPad Software, USA).

## Results

### Ketamine induces A549 cells apoptosis in a dose‐dependent manner

**Figure 1:**
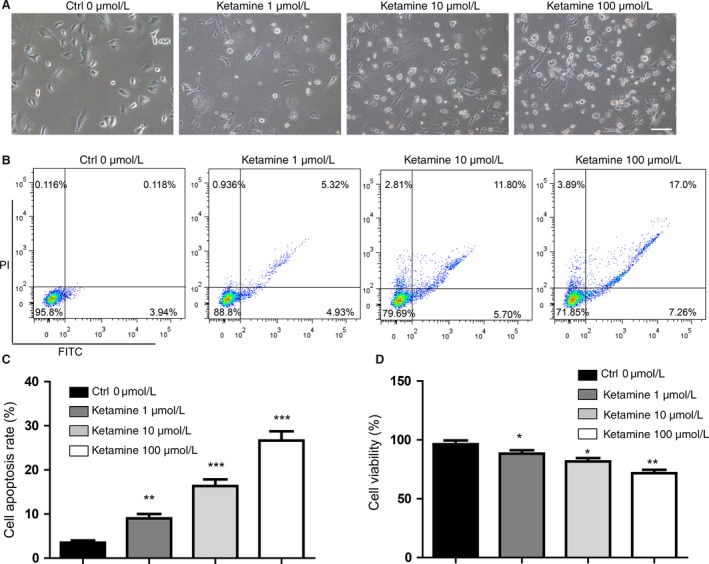
Ketamine induces apoptosis of A549 cells in a dose‐dependent manner. (A) Morphology of cells treated with increasing concentration of ketamine. Scale bar represents 100 *μ*m. (B) FACS analysis for detecting the increasing apoptosis proportion of cells treated by increasing concentration of ketamine. (C) Statistics of apoptosis proportion of cells treated by ketamine. (D) Trypan blue staining indicated the repression of cell viability in cells treated by ketamine. Data represent mean ± SD of three independent experiments (*n *=* *3, *P <* *0.05, **P <* *0.01, ***P <* *0.001).

To evaluate whether ketamine induces apoptosis in A549 cells, the cells were exposed to ketamine (0, 1, 10, and 100 *μ*mol/L) for 24 h. We found that 10 and 100 *μ*mol/L ketamine induced a significant degree of apoptosis in A549 cells compared with the control group (Fig. 1A). FACS analysis confirmed that ketamine significantly induced the apoptosis of A549 cells at concentrations of 10 and 100 *μ*mol/L (Fig. 1B and C). Assessing cell viability by trypan staining showed that 10 and 100 *μ*mol/L ketamine induced more than 10–20% of the cells to die (Fig. 1D).

### Ketamine upregulates the expression of CD69 in A549 cells

**Figure 2:**
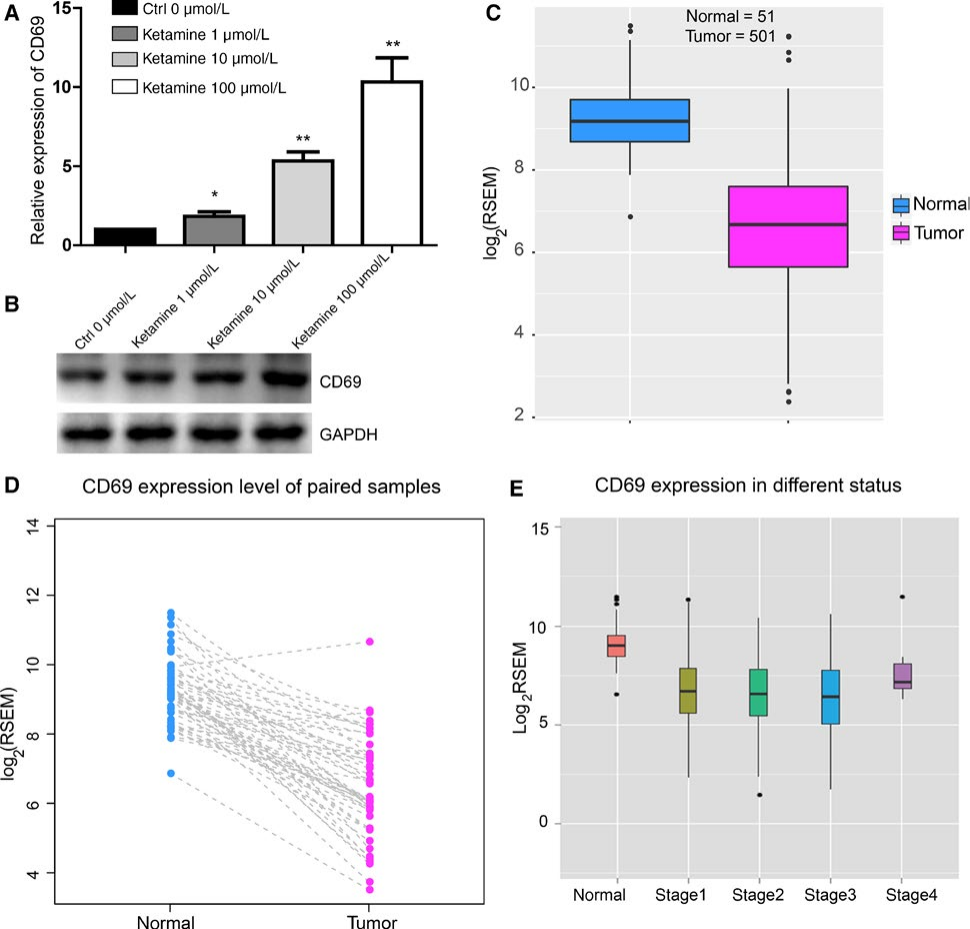
Effects of ketamine on cell growth inhibition of A549 cells. (A) Detection of CD69 mRNA level in A549 cells treated with 0, 1, 10, and 100 *μ*m ketamine for 24 h, respectively. Data represent mean ± SD of three independent experiments (*n *=* *4, *P <* *0.05, **P <* *0.01, ***P <* *0.001). (B) The CD69 level detected by western blot analysis. (C) Expression of CD69 in the normal (*n* = 51) and cancer (*n* = 501) tissues. *P* = 1.31e–29. (D) CD69 expression was lower in the cancer tissues compared with the normal tissues in same patients. *P* = 7.90e−17. (E) RSEM analysis showed downregulation of the expression level of CD69 in cancer tissues compared with normal tissues and associated with the T stage.

We further found that CD69 could be upregulated by ketamine in A549 cells at both the mRNA (Fig. 2A) and protein levels (Fig. 2B). The expression of CD69 was clearly augmented as the concentration of ketamine was increased (Fig. 2A and B). We therefore analyzed data from lung cancer patient samples in The Cancer Genome Atlas (TCGA) database and found that CD69 was significantly decreased in cancer samples compared with normal tissues (Fig. 2C). Paired comparison of the normal and cancer samples also indicated the downregulation of CD69 in the cancers (Fig. 2D). The tumor, node, metastases (TNM) stage system is used to classify patients for clinical treatment or participation in clinical trials. We found that the expression level of CD69 was associated with the T stage, especially the T1, T2, and T3 stages, which indicated that low expression of CD69 was associated with an increase in tumors (Fig. 2E).

### Overexpression of CD69 induces apoptosis of A549 cells

**Figure 3:**
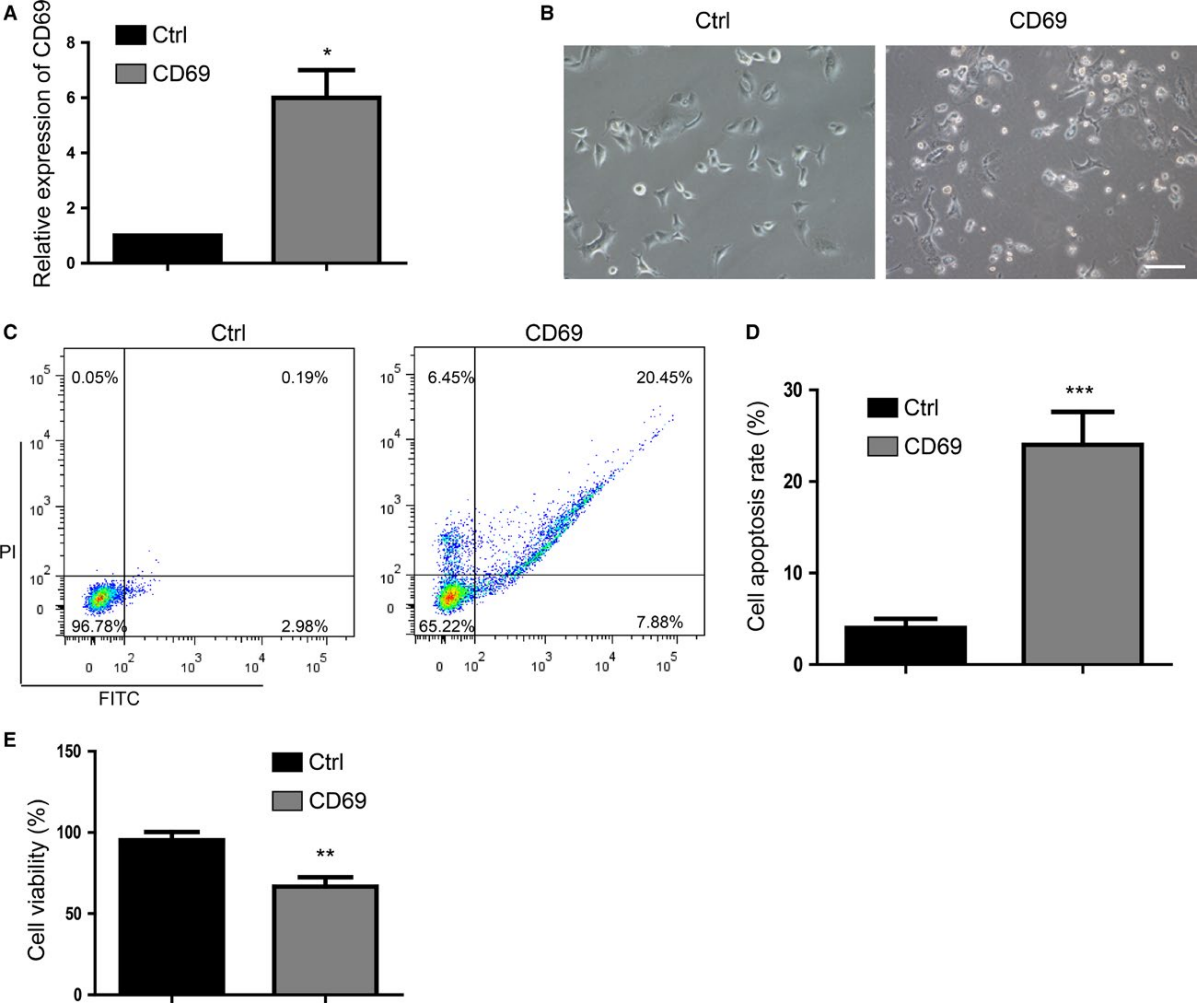
CD69 induces the cell apoptosis in A549 cells. (A) Detection of CD69 mRNA level in control group and CD69 overexpression group by qPCR. (B) The morphology of cells observed by inverted phase‐contrast microscope. Scale bar represents 100 *μ*m. (C) FACS analysis of cell apoptosis and (D) statistics of cell apoptosis rate in CD69 overexpression group. (E). Cell viability of the cells overexpressed with CD69 and control group. Values are expressed as means ± SD (*n *=* *3). *P <* *0.05, **P <* *0.01, ***P < 0.001.

We then focused our attention on the apoptosis‐regulatory function of CD69. To confirm that CD69 crucially contributed to apoptosis, we transfected A549 cells with Fugw‐CD69 to overexpress CD69. CD69 levels were significantly higher in transfected cells than in the control group (Fig. 3A). There were more apoptotic cells, which were small and showed shrinkage, in the CD69 overexpressing group (Fig. 3B). As shown in Figure 3C, overexpression of CD69 significantly induced apoptosis compared with the control group, as assessed by FACS. The regulatory effect of CD69 overexpression on the apoptosis rate and viability of A549 cells is shown in Figure 3D and E.

### CD69 mediated ketamine‐induced apoptosis

**Figure 4:**
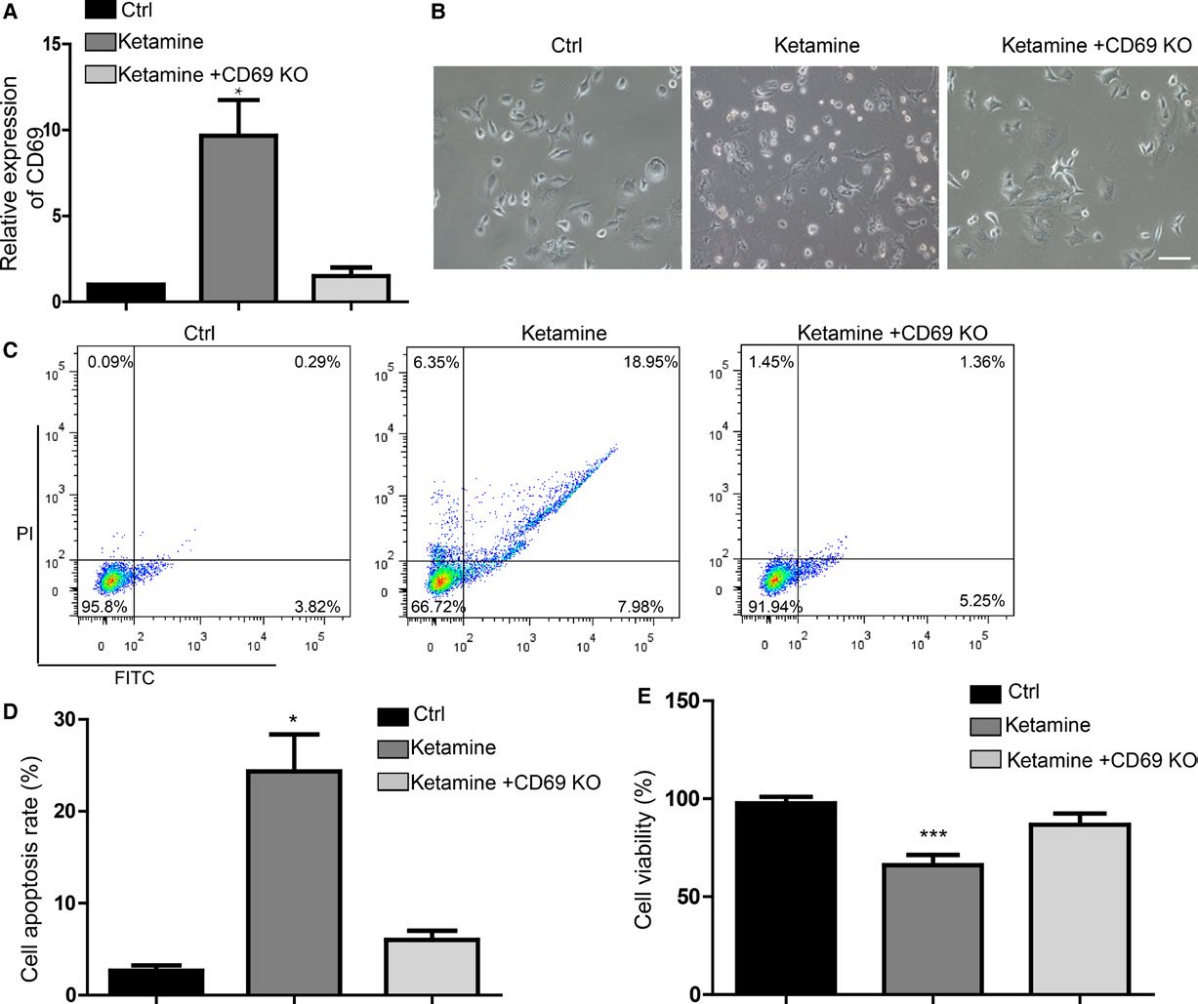
Knockdown of CD69 alleviates the effect of ketamine on inducing apoptosis. (A) Detection of relative expression level of CD69 in rescue experiment. CD69 KO means CD69 knockdown. (B) Morphology of cell apoptosis showed that knockdown of CD69 significantly blocked the function of ketamine on upregulating the CD69. Scale bar represents 100 *μ*m. (C) FACS analysis of cell apoptosis proportion in rescue experiment. (D) Statistics of cell apoptosis rate. (E) Cell viability detection. Data represent mean ± SD for three independent experiments. *P <* *0.05, **P <* *0.01, ***P <* *0.001.

To determine whether CD69 was the downstream target of ketamine, we performed rescue experiments. We treated the A549 cells with 10 *μ*mol/L ketamine and also downregulated the expression of CD69. We found that knockdown of CD69 significantly blocked the ketamine‐induced upregulation of CD69 (Fig. 4A). We then found that ketamine‐treated A549 cells with knockdown of CD69 showed similar cell morphology to the control group (Fig. 4A). The knockdown of CD69 could block the cell apoptosis induced by ketamine (Fig. 4C and D). The knockdown of CD69 also rescued the cell viability repressed by ketamine (Fig. 4E).

## Discussion

Our study demonstrates that ketamine/CD69 pathways control the apoptosis of LUAD cells. These effects of ketamine are due to the promotion of CD69 expression. The exposure of lung carcinoma cells to ketamine results in an increased level of CD69 expression. These changes in gene expression explain the proapoptotic effect of ketamine in human lung adenocarcinoma cells.

Apoptosis, a tightly regulated cell suicide program, plays a vital role in the development and maintenance of tissue homeostasis by eliminating unnecessary and unwanted cells 21. Apoptosis is also a critical pathological change involved in human cancer. Cancer cells possess the ability to escape apoptosis as they can impair apoptotic signaling, and this facilitates tumor development and metastasis. In cancer therapy, the inhibition of cancer cell proliferation cannot completely eliminate tumors but rather postpones the progression of tumorigenesis. By contrast, the induction of cancer cell apoptosis is a major goal for oncologic treatment, as it could reduce tumor size or even eliminate tumors 13. Therefore, the identification of reagents that can induce cancer cell apoptosis has important clinical implications. Activating the apoptotic machinery in tumor cells represents a potential and promising avenue in cancer treatment. In our study, we investigated the effects of ketamine on LUAD cell apoptosis, and the results showed that ketamine can induce cancer cells apoptosis. This induction of apoptosis has also been confirmed in neurons, pancreatic cancer cells, and HepG2 cells 7, 11, 22. By contrast, in breast cancer cells, ketamine could inhibit cancer growth by promoting antiapoptotic effects 23. Recent study showed that ketamine caused the dose‐dependent, statistically significant increase of neuroapoptosis 24. However, low doses of ketamine protect against inflammatory pain‐induced neurotoxicity 25. Our studies suggested that ketamine might be the novel efficient anti‐cancer drug even at the low concentration of 10 *μ*mol/L. We further need to detect the exact dosage of ketamine on repressing the lung cancer in vivo, which can indicate the safe scope of ketamine usage for patients in future.

An increasing number of studies have shed light on the mechanism of action of NMDA antagonist. Stepulak et al. 26 reported that dizocilpine inhibits the ERK1/2 pathway in lung carcinoma cells and reduces the phosphorylation of CREB and the expression of CREB‐regulated genes, which inhibits cell cycle progression and proliferation in cancer cells. Meanwhile, Lee et al. 22 showed that S‐(+)‐ketamine can induce apoptotic insults in human HepG2 cells via a Bax–mitochondria–caspase protease pathway. In the study described in this article, we found that ketamine promoted the expression of CD69. CD69 is the marker of activated cells, such as lymphocytes and NK cell 27. Previous studies showed that hepatic CD69+ Kupffer cells repressed proliferation of Ag‐nonspecific and OVA‐specific CD4 T cell through TGF‐*β*1 28, 29. In T cell, CD69 controls the differentiation through the interaction with galectin‐1 30.

We found that CD69 was downregulated in the cancer tissues compared with normal tissues. There was downregulation of CD69 in the malignant advanced tumor. These results indicated the critical correlation between the expression of CD69 with the cancer genesis and development. We further found that overexpression of CD69 intensified the proapoptotic effect of ketamine on cancer cells. The present results indicate that ketamine induces lung carcinoma cell apoptosis by activating CD69 gene transcription.

This novel finding may thus represent an important link between CD69 and NSCLC, and it highlights the critical role of CD69 in apoptosis. In past studies, CD69 was detected on eosinophils and resulted in eosinophil apoptosis by transducing a Bcl‐2‐dependent death signal 18, 31. CD69 is constitutively expressed on monocytes, platelets, and some peripheral lymphocyte populations, including mucosal intestinal cells and bronchoalveolar lavage cells 17. CD69 was also shown to induce apoptosis in human monocytes/macrophages 17. CD69‐mediated apoptosis can be explained by the association with an N‐terminal fragment of calreticulin at the cell surface 32, which may be involved in the CD69‐mediated mechanism of ketamine‐induced apoptosis.

There are certain limitations in this study. Future work should include investigation of the effect of ketamine on solid tumors or animals in vivo. Our study showed that ketamine can trigger apoptotic insults after exposure for 24 h, may be prolonged exposure could be of more significance in clinical practice when ketamine used for longer periods such as for treatment of postoperative pain, or as a sedative in the intensive care unit.

## Conclusion

In conclusion, this study shows that ketamine induces cancer cells apoptosis. In addition, it provides insight into the role of the CD69 on growth inhibition by ketamine in NSCLC. Our findings reveal prospects for ketamine in cancer therapy. Meanwhile, CD69 may serve as a new therapeutic target of NSCLC.

## Conflict of Interest

None declared.
